# The challenges of treating tracheobronchitis in a laryngectomee due to nontypeable *Haemophilus influenzae*: a case report

**DOI:** 10.1186/s13256-018-1764-2

**Published:** 2018-08-20

**Authors:** Itzhak Brook

**Affiliations:** Department of Pediatrics and Medicine, Georgetown University School of Medicine, Washington DC, 20007 USA

**Keywords:** Tracheobronchitis, Laryngectomee, *Haemophilus influenzae*, Prevention

## Abstract

**Background:**

Laryngectomees run the risk of developing severe respiratory tract infections especially during the winter and when they do not wear a stoma cover. A case of severe tracheobronchitis in a laryngectomee is presented that illustrates the risks and difficulties encountered in managing this infection in a neck breather.

**Case presentation:**

A 76-year-old Caucasian man, a laryngectomee, presented with bacterial tracheobronchitis and conjunctivitis due to beta-lactamase-producing nontypeable *Haemophilus influenzae*. He was febrile (38.9 °C; 102.0 F), and had repeated episodes of hypertension. He was treated with levofloxacin 500 mg/day, ciprofloxacin eye drops, acetaminophen, and guaifenesin. Humidification of his trachea and the airway was sustained by insertions of saline into the stoma as well as breathing humidified air. The main challenge was to maintain the patency of his airway as the mucus was very dry and viscous and tended to stick to the walls of his trachea and the stoma. His condition improved within 7 days and he had a complete recovery.

**Conclusions:**

Maintaining the patency of the airway in laryngectomees who suffer from lower respiratory tract infection is of utmost importance as the mucus can be very dry and viscous and can stick to the walls of the trachea and the stoma.

## Background

Laryngectomees run a high risk of developing severe respiratory tract infections. Following laryngectomy the tracheal epithelium becomes directly exposed to the relatively cold and dry ambient air entering the tracheostoma [1, 2]. This can cause: drying of the mucus, which makes it more viscous; reduction of ciliary activity that causes impaired mucociliary clearance [2–4]; and tracheal epithelium damage (loss of ciliated cells, goblet cell hyperplasia, and excessive mucus production and metaplasia) [4].

Severe pulmonary infections (tracheobronchitis and pneumonia) in laryngectomees are more frequent in the wintertime and the accompanying tracheal crusting often requires antibiotic treatment or even hospitalization [5]. Tracheobronchitis in laryngectomees was described as a “suffocating” respiratory infection because of the difficulties in maintaining a patent airway in these patients [6, 7].

A case of severe tracheobronchitis in a laryngectomee is presented that illustrates the risks and difficulties encountered in managing this infection in a neck breather.

## Case presentation

A 76-year-old Caucasian man who underwent laryngectomy 10 years earlier, presented with fever (38.9 °C; 102.0 °F), increased sputum production, and purulent conjunctivitis. These symptoms emerged gradually over a period of 48 hours. He noted increasing difficulty in coughing out his sputum that became brownish and viscous. He had been wearing a heat and moisture exchanger (HME) filter that covered his stoma and spoke through a tracheoesophageal voice prosthesis. The symptoms started a day after a very cold weather spell with temperatures of −7 to −1 °C (19–31 °F). He had to remove his HME on several occasions for extended periods of time to enable him to breathe when he walked outside his home.

His past medical history included hypopharyngeal squamous cell carcinoma which was treated with intensity-modulated radiotherapy (IMRT) 12 years earlier. A recurrence of the cancer 2 years later required laryngectomy. He had no signs of tumor recurrence since then. He also suffered from paroxysmal hypertension, diverticulitis, and migraines.

He was vaccinated with the current Influenza virus vaccine 3 month earlier. He had also received a pneumococcal polysaccharide vaccine (PPSV23) 2 years earlier.

He was in mild respiratory distress especially when coughing. He had coughing spells and expectorated green-brown dry and viscous sputum. A physical examination revealed bilateral purulent conjunctivitis and auscultation of his lungs revealed coarse rhonchi and no crepitations. No lymphadenopathy was noted. The results of the rest of the physical and neurological examinations were within normal limits. A chest X-ray was normal.

Sputum and conjunctival culture grew heavy growth of beta-lactamase-producing nontypeable *Haemophilus influenzae* (NTHi) that was susceptible to levofloxacin and amoxicillin- clavulanate. A FilmArray® Respiratory Panel 2 (RP2) polymerase chain reaction (PCR) system test did not detect 14 viruses (adenovirus, coronavirus HKU1, coronavirus NL63, coronavirus 229E, coronavirus OC43, human rhinovirus/enterovirus, human metapneumovirus, influenza A, influenza B, parainfluenza virus 1, parainfluenza virus 2, parainfluenza virus 3, parainfluenza virus 4, respiratory syncytial virus) and four bacteria (*Bordetella pertussis, Bordetella parapertussis, Chlamydophila pneumoniae, Mycoplasma pneumoniae*).

He was treated with orally administered levofloxacin 500 mg/day, ciprofloxacin eye drops, acetaminophen, and guaifenesin. Humidification of his trachea and the airway was maintained by repeated insertions of 3–5 cc respiratory saline into the stoma at least once an hour and by breathing humidified air.

The main challenge was to maintain a patent airway as the mucus was very dry and viscous and tended to stick to the walls of his trachea and the stoma. The mucus had to be repeatedly expectorated by vigorous coughing and by manual removal from the upper part of his trachea and stoma.

He experienced repeated episodes of sustained elevated blood pressure (up to 210/110) and tachycardia (112/minute). This was managed by administration of clonidine 0.1 mg as needed (1–2/day).

His fever started to decline 48 hours after antimicrobial therapy was started. The conjunctivitis improved within 36 hours. The sputum production declined and became less viscous over time, but persisted for 5 days.

Antimicrobial therapy was discontinued after 7 days.

His condition improved and he had a complete recovery in 7 days. He was seen in the clinic every 2 months and showed no recurrence of his infection for the following 8 months. He received vaccination for *H. influenzae* B and Prevnar 13® (pneumococcal conjugate vaccine; PCV13) 4 weeks after his recovery.

## Discussion

This case report described the occurrence of a serious bacterial tracheobronchitis due to *H. influenzae* in a laryngectomee. It illustrates the difficulties in managing the airway access in laryngectomees because of the increased mucus production that can block them. It also highlights the need to recognize the potential of bacterial infection in this population.

NTHi is a known cause of community-acquired pneumonia in adults [8] and may be associated with severe disease and high mortality in higher risk populations. Although bacteremia that complicates *H. influenzae* pneumonia occurs occasionally [9], disseminated infections following NTHi bacteremia are uncommon except in newborns and immunocompromised patients. *H. influenzae* was recovered from 6 of 27 (22%) tracheal aspirates of children with tracheostomy who had pneumonia [10], and in 4 of 14 (29%) children with bacterial tracheitis [11].

The stoma in neck breathers allows the inhaled air to bypass the natural defenses (nasal hair and mucus membranes) of the upper airway that filter out dust and bacteria. Wearing an HME that covers the stoma can provide important benefits [1, 2]. The HME filter serves as a stoma cover and creates a tight seal around the stoma. In addition to filtering dust and other larger airborne particles, HMEs preserve some of the moisture and heat inside the respiratory tract and prevent their loss and adds resistance to the airflow [1, 2]. HME filters assist in restoring the temperature, moisture, and cleanliness of the inhaled air to the same condition as before laryngectomy.

The number of bronchitis, tracheobronchitis, and pneumonia episodes as well as mortality due to these infections in non-HME users was found to be three times higher than in HME users [12]. Laryngectomees especially those who do not wear an HME or have uncovered stoma are therefore at a higher risk for lower respiratory infections.

The exposure of our patient to cold air without an HME most likely compromised his airway and led to the development of the infection.

Paroxysmal hypertension is a known complication following head and neck radiation, and can be attributed to damage to the carotid artery baroreceptors [13]. Aggravation of this condition during our patient’s illness further complicated his condition.

Treatment of tracheobronchitis in laryngectomees is more challenging and requires keeping the stoma open by manually removing accumulated mucus that can dry out and clog it, keeping the excessive sputum moist by breathing humidified air and inserting saline as needed, coughing out or suctioning accumulated sputum, keeping the patient well hydrated, and removing the HME during the illness or prior to coughing to prevent blocking it with the coughed out sputum.

## Conclusions

Laryngectomees are at a higher risk of developing lower respiratory tract infections especially in the winter and when not wearing an HME. Maintaining the patency of the airway is of utmost importance as the mucus can be very dry and viscous and can stick to the walls of the trachea and the stoma. The risk of acquiring these infections can be reduced by: getting vaccinated for respiratory pathogens that include *Streptococcus pneumoniae, H. influenzae*, and the influenza viruses; washing hands before any stoma care; wearing an HME at all times; maintaining adequate respiratory tract humidification; and avoiding hypothermia or inhaling cold air.
